# Danshensu alleviates cardiac ischaemia/reperfusion injury by inhibiting autophagy and apoptosis *via* activation of mTOR signalling

**DOI:** 10.1111/jcmm.12883

**Published:** 2016-07-07

**Authors:** Guanwei Fan, Jiahui Yu, Patrick Fordjour Asare, Lingyan Wang, Han Zhang, Boli Zhang, Yan Zhu, Xiumei Gao

**Affiliations:** ^1^ State Key Laboratory of Modern Chinese MedicineTianjin University of Traditional Chinese MedicineTianjinChina; ^2^ Ministry of Education Key Laboratory of Pharmacology of Traditional Chinese Medical FormulaeTianjin University of Traditional Chinese MedicineTianjinChina; ^3^ Institute of Traditional Chinese Medicine ResearchTianjin University of Traditional Chinese MedicineTianjinChina

**Keywords:** Danshensu, I/R injury, autophagy, apoptosis, mTOR

## Abstract

The traditional Chinese medicine Danshensu (DSS) has a protective effect on cardiac ischaemia/reperfusion (I/R) injury. However, the molecular mechanisms underlying the DSS action remain undefined. We investigated the potential role of DSS in autophagy and apoptosis using cardiac I/R injury models of cardiomyocytes and isolated rat hearts. Cultured neonatal rat cardiomyocytes were subjected to 6 hrs of hypoxia followed by 18 hrs of reoxygenation to induce cell damage. The isolated rat hearts were used to perform global ischaemia for 30 min., followed by 60 min. reperfusion. Ischaemia/reperfusion injury decreased the haemodynamic parameters on cardiac function, damaged cardiomyocytes or even caused cell death. Pre‐treatment of DSS significantly improved cell survival and protected against I/R‐induced deterioration of cardiac function. The improved cell survival upon DSS treatment was associated with activation of mammalian target of rapamycin (mTOR) (as manifested by increased phosphorylation of S6K and S6), which was accompanied with attenuated autophagy flux and decreased expression of autophagy‐ and apoptosis‐related proteins (including p62, LC3‐II, Beclin‐1, Bax, and Caspase‐3) at both protein and mRNA levels. These results suggest that alleviation of cardiac I/R injury by pre‐treatment with DSS may be attributable to inhibiting excessive autophagy and apoptosis through mTOR activation.

## Introduction

Acute myocardial infarction (MI) is a major cause of death worldwide 1 and often occurs under various clinical conditions, such as thrombolysis, percutaneous coronary angioplasty, organ transplantation and coronary bypass 2. Prompt reperfusion remains one of the most effective methods in the treatment of acute MI 1. However, reperfusion treatment has a potential risk of worsening tissue damage after ischaemia by the phenomenon of ischaemic/reperfusion (I/R) injury, which can accelerate the deterioration of cardiac function 3.

Increasing evidence suggests that autophagy is activated during various pathologic conditions in the heart and vasculatures 4, including heart failure 5, hypertrophy 6, chronic and acute myocardial ischaemia 7 and atherosclerosis 8. Autophagy plays an essential role in stress adaption in myocardial injury through removal of protein aggregates and damaged organelles, and providing ‘emergency energy’ to maintain cell survival. Further, autophagy can provide a protective response during acute myocardial ischaemia. However, excessive activation of autophagy can induce extensive degradation of cytosolic essential proteins and organelles leading to cell death 9, 10. For example, myocardial I/R injury causes excessive autophagy, resulting in cytotoxic cell death 11, 12. Thus, prevention of I/R‐induced excessive autophagy may reduce cardiomyocyte death and preserve cardiac function.

Apoptosis, a form of programmed cell death, can occur in a wide range of physiological and pathological situations. It is characterized by cell shrinkage, programmed DNA degradation, increased cytoplasmic cytochrome C release and activation of caspases. It is known that I/R activates apoptosis in the heart 13, 14, 15. The B‐cell lymphoma (Bcl)‐2 family proteins are important regulators of apoptosis in I/R 16. Antiapoptotic members such as Bcl‐2 promote survival by inhibiting the function of the proapoptotic Bax proteins. Bax is also activated in myocytes in response to oxidative stress and during I/R 17, 18. Furthermore, activated Caspase‐3 in the mitochondrial apoptotic pathway can lead to loss of ATP and reactive oxygen species (ROS) generation. During the I/R period, increased ROS can upregulate the expression of the autophagy‐ promoting protein Beclin‐1, and microtubule associated protein light chain 3 (LC3), and p62 leading to autophagy 19, 20. There is now increasing evidence of potential cross‐talk between the autophagy and apoptosis pathways 21. For example, Beclin‐1 forms complexes with Bcl‐2, while Bcl‐2 is a negative regulator of autophagy *via* an interaction with Beclin‐1 22. Thus, overexpression of Bcl‐2 may protect against I/R injury by blocking apoptosis as well as preventing activation of autophagy by inhibiting Beclin‐1. Therefore, Bcl‐2 is able to inhibit Beclin‐1 expression by interacting with Beclin‐1to form Beclin‐1:Bcl2 complex. Thus, Beclin‐1 executes autophagy once Bcl‐2 dissociates from Beclin:Bcl‐2 complex. That being said, Beclin‐1 does not inhibit Bcl‐2 but rather serves as a key structural motif that enables Bcl‐2 to not only function as an anti‐apoptotic protein but also as an anti‐autopahgic protein.

The mammalian target of rapamycin (mTOR) pathway is a key regulator of cell growth and proliferation 23, and mTOR inhibition results in induction of autophagy. Overexpression of mTOR in the heart can provide substantial cardioprotection against I/R injury 24. However, the relationships between mTOR signalling pathways that regulate autophagy and those that regulate apoptosis remain incompletely understood.

Danshensu (DSS), a water‐soluble constituent of *Salvia miltiorrhiza* (Danshen), is well‐recognized for its cardiovascular activity. DSS was previously reported to protect the heart against ischaemia/reperfusion (I/R) injury by reducing ROS generation and inhibiting cell apoptosis 25, 26, 27. However, the effects of DSS on the autophagy and apoptosis pathways during I/R injury are unknown. In the present study, we investigated the protective effects of DSS against cardiac I/R injury, and the potential roles of the autophagic and apoptotic pathways in models of cardiomyocytes and isolated rat hearts. Rapamycin (Rap), an inhibitor of mTOR was used to irreversibly inhibit mTOR to further examine whether the DSS inhibition of autophagy and apoptosis was actually due to activation of mTOR.

## Materials and methods

### Materials

Danshenshu (98%, purity) was purchased from Chinese Institute for Drug and Biological Product Control (Beijing, China). The kits for determination of creatine kinase (CK) and lactate dehydrogenase (LDH) were obtained from Jiancheng Bioengineering Institute (Nanjing, China). The BCA Protein Assay Kit was from Thermo Scientific (Waltham, MA, USA). Transcriptor First Strand cDNA Synthesis Kit and FastStart Universal SYBR Green Master Mix Kit were obtained from Roche (Basel, Switzerland). Primary antibodies against mTOR (ab32028), phospho‐mTOR (Ser2448) (ab109268), phospho‐S6K1(T389) (ab126818), phospho‐S6(S235/S236) (ab12864), p62 (ab56416), Beclin1 (ab51031), Bcl‐2 (ab59348), Bax (ab32503) and Caspase‐3 (ab44976) were purchased from Abcam (Cambridge, MA, USA). LC3B (L8918) was purchased from Sigma‐Aldrich (St. Louis, MO, USA). GAPDH (#5174) was purchased from Cell Signaling Technology (Danvers, MA, USA).

### Animal and ethics

Male Sprague‐Dawley rats (300 ± 20 g) were purchased from Beijing Vital River Lab Animal Technology Co. Ltd (Beijing, China). Ambient temperature (22 ± 2°C) and relative humidity (60 ± 5%) were maintained. The rats were allowed to acclimatize to the housing facilities before the experiments and were provide with standard diet and water *ad libitum*. This study was performed in accordance with the recommendations in the Guidance for the Care and Use of Laboratory Animals issued by the Ministry of Science and Technology of China and the protocol approved by the Laboratory Animal Ethics Committee of Tianjin University of Traditional Chinese Medicine (Permit Number: TCM‐LAEC2014004).

### Harvest and culture of neonatal rat cardiomyocytes

Cardiomyocytes were prepared from 1 to 3 days old neonatal Sprague‐Dawley rats of both sexes. The hearts were removed quickly and washed three times with cold PBS. Epicardial adipose tissue and aortas were discarded and the left heart tissues were minced with scissors into 1 mm^3^ fragments. The fragments were digested with PBS containing 0.0625% (w/v) trypsin and 0.1% (w/v) collagenase II by gentle shaking at 37°C for 5 min. each time. Digestion was repeated 5–8 times in total with new digestion buffer. Digestion was ceased with the addition of medium containing 10% (v/v) foetal bovine serum. The cell suspension was subsequently filtered with a 200‐mesh sieve and centrifuged at 174 × g for 10 min. The obtained cell pellet was resuspended and incubated for 90 min. at 37°C with basic medium containing 10% (v/v) foetal bovine serum to allow fibroblast adhesion. Non‐adherent cells were harvested and plated in a culture flask. This method yielded purer cardiomyocyte cultures. Bromodeoxyuridine (0.1 mM) was added to the medium for the first 2 days to inhibit the growth of fibroblasts. After 3 days of culture, cardiomyocytes could be used for subsequent experiments 28.

### Hypoxia/reoxygenation injury

**Figure 1:**
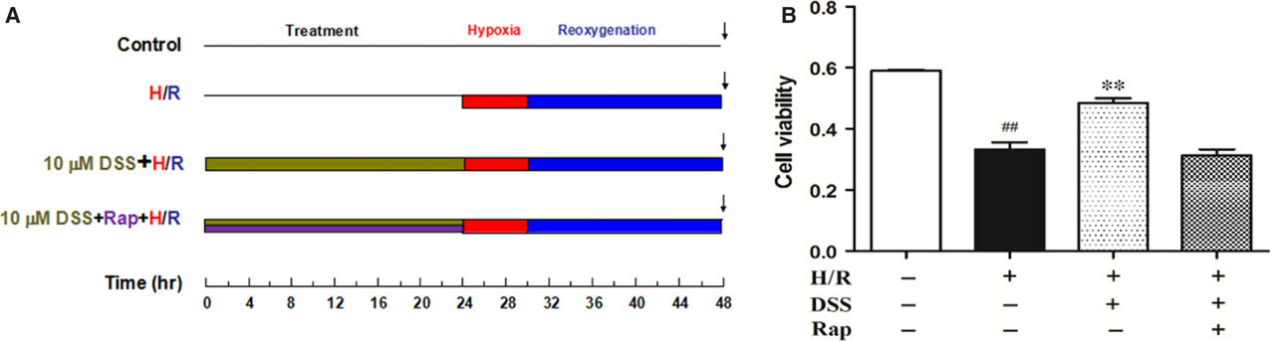
Experimental protocol and effects of DSS treatment on cellular viability. (**A**) Hypoxia/reoxygenation (H/R) injury protocol. Control: cells were cultured constantly under normoxic condition. H/R: after normoxic condition cultured for 24 hrs, cells received 6 hrs of hypoxia and 18 hrs of reoxygenation. Drug treatments: after pre‐treatment with 10 μM DSS or 10 μM DSS + 100 nM Rap for 24 hrs, H/R was simulated as previously described. (**B**) Cell viability of cardiomyocytes. Data are expressed as the mean ± S.D., *n* = 4. ^##^ *P* < 0.01 *versus* Control group. **P < 0.01 *versus* H/R group.

To simulate I/R injury, we performed hypoxia/reoxygenation (H/R) with cardiomyocytes. After pre‐treatment with 10 μM DSS or 10 μM DSS + 100 nM Rap for 24 hrs, H/R was simulated as previously described 29. The cultured cardiomyocytes were incubated in glucose‐free DMEM (pH 6.8) and then placed into a hypoxia chamber (StemCell Technologies, San Diego, CA, USA). The chamber was flushed with 95% (v/v) N_2_ and 5% (v/v) CO_2_ at a flow rate of 15 l/min. for 10 min. and maintained at 37°C. After 6 hrs hypoxia, reoxygenation was accomplished by replacing the medium to DMEM containing 4.5 mM glucose (pH 7.4) and subsequent incubation in a CO_2_ incubator (5% (v/v) CO_2_‐95% (v/v) air) for 18 hrs (Fig. 1).

### Assessment of cell viability with CCK‐8 assay

Cell viability was assessed with the cell count kit‐8 (CCK‐8; Dojindo, Kumamoto, Japan) according to the manufacturer's instructions. Cells (2.5 × 10^4^/well) were seeded in 96‐well plates and applied to various culture conditions, including control, H/R, and 10 μM DSS, or 10 μM DSS + 100 nM Rap treatments, as described above. At the end of each treatment, the culture medium was replaced with 100 μl CCK‐8 solution (DMEM/F12:CCK‐8 = 10:1). The absorbance at 450 nm was then measured after approximately 2–4 hrs recovery.

### Evaluation of autophagy flux

mRFP‐GFP‐LC3‐transfected cardiomyocytes were visualized by confocal microscopy. To quantify the number of puncta, mRFP‐GFP‐LC3 transfected cells were seeded in a dish. Thirty‐six hours after adenovirus transduction, the cells were subjected to H/R injury as described above. The cells were then analysed by confocal microscopy. The number of puncta per cell was determined using Image Pro Plus software (Media Cybernetics, Inc., Rockville, MD, USA).

### Isolated rat heart perfusion and Langendorff procedure

24 Rats were anesthetized with sodium pentobarbital (50 mg/kg i.p.). A thoracotomy was then performed and hearts were rapidly excised into an ice‐cold Krebs‐Henseleit solution (KHs). After removal of the lungs and surrounding tissues, the aorta was attached to the perfusion device where hearts were perused at a constant retrograde flow, according to the method of Langendorff with a non‐recirculated KHs of the following composition (mM): NaCl 118, KCl 4.7, CaCl_2_ 1.25, MgSO_4_ 1.2, KH_2_PO_4_ 1.2, glucose 11.0, and NaHCO_3_ 25.0, gassed with carbogen (95% O_2_ and 5% CO_2_) at 37°C to obtain a physiological pH of 7.4. The hearts were perfused with constant pressure of approximately 65 mmHg and left for 20 min. to permit recovery of function and stabilize the rhythm. The LV end diastolic pressure was maintained at 5–10 mmHg, and coronary flow (CF) was measured by collecting the effluent dripping from the heart perfusate 30.

Cardiac function was evaluated by measuring left ventricular developed pressure (left ventricle end systolic pressure minus left ventricle end diastolic pressure; LVDP = LVSP − LVEDP), maximal and minimum rate of pressure development (±dp/dt max), and heart rate (HR). LVDP, ± dp/dt max, and HR were calculated from the LV pressure curve. These parameters were recorded continuously on a computer using Powerlab data acquisition system (8SP Chart 5 software; A.D. Instruments, Castle Hill, Australia) 30.

Hearts were then randomly assigned to the following groups: (*i*) Control group (hearts perfused with KH buffer for 120 min.); (*ii*) I/R group (hearts that were allowed to stabilize for 30 min. prior to being subjected to 30 min. global ischaemia achieved by discontinued KHs perfusion, followed by 60 min. reperfusion); (*iii*) DSS group (hearts perfused with DSS 10 μM for 10 min. after 20 min. of stabilization, global ischaemia for 30 min., followed by 60 min. reperfusion); and (*iv*) DSS + Rap group (hearts perfused with Rap 100 nM for 5 min. and later perfused with DSS 10 μM for 10 min. after 20 min. of stabilization, global ischaemia performed for 30 min., followed by 60 min. reperfusion).

### Creatine kinase and lactate dehydrogenase assay

As additional markers of myocardial injury, CK and LDH were determined from the coronary effluent that was collected at the end of reperfusion, as previously described 31. Creatine kinase and LDH levels were assayed spectrophotometrically using commercial kits, and levels expressed in units per litre of coronary effluents.

### Real time quantitative RT‐PCR analysis

After the 60 min. reperfusion period, total RNA was isolated from the LV myocardial tissue using Trizol reagent according to the standard protocol. Total RNA was reverse transcribed in 20 μl of a reaction mixture that contained reverse transcriptase, 5× reaction buffer, RNase inhibitor, dNTP mix and random hexamer primers, using the Transcriptor First Strand cDNA Synthesis Kit (Roche), run at 25°C for 10 min., 50°C for 1 hr, 85°C for 5 min. and then stored at 4°C. Quantification of gene expression was performed with real‐time quantitative RT‐PCR (Real‐Time PCR System; Bio‐Rad, Hercules, CA, USA) using the Fast Start Universal SYBR Green Master Mix Kit (Roche) and specific primers (Table 1). The 25 μl total PCR reaction volumes consisted of specific primers (0.2 μM final concentrations), 50 ng of cNDA template, 2× SYBR Green master mix and PCR‐grade water. Amplification was performed under the following cycle conditions: 10 min. at 95°C, 40 cycles of 15 sec. denaturation at 95°C, and annealing at 60°C for 40 sec. The threshold cycle (CT) value was determined and the relative mRNA expression of the genes was calculated as follows: 2^−ΔΔCT^ with ^ΔΔ^CT = CTGAPDH–CT gene of interest. Negative control reactions were run without cDNA. All analyses were performed in triplicate.

**Table 1:** Primer sequences for RT‐PCR analysis

Primers	Sequences (5′–3′)
mTOR	Forward: TGCTGGTGTCCTTTGTGAAG
	Reverse: TTGTGCTCTGGATTGAGGTG
p62	Forward: GCTGCCCTGTACCCACATCT
	Reverse: CGCCTTCATCCGAGAAAC
LC3	Forward: ATCATCGAGCGCTACAAGGGTGA
	Reverse: GGATGATCTTGACCAACTCGCTCAT
Beclin1	Forward: TTCAAGATCCTGGACCGAGTGAC
	Reverse: AGACACCATCCTGGCGAGTTTC
Bcl‐2	Forward: GAGCGTCAACAGGGAGATGT
	Reverse: CAGCCAGGAGAAATCAAACAG
Bax	Forward: TTGCTACAGGGTTTCATCCA
	Reverse: TGTTGTTGTCCAGTTCATCG
Caspase‐3	Forward: AGCTGGACTGCGGTATTGAG
	Reverse: GGGTGCGGTAGAGTAAGCAT
GAPDH	Forward: ATGATTCTACCCACGGCAAG
	Reverse: CTGGAAGATGGTGATGGGTT

### Western blotting analysis

After 60 min. reperfusion, great vessels and atria of the heart were trimmed away and the ventricles were cut open, weighed and then snap‐frozen and stored at −80°C until use. At the end of reoxygenation, proteins from cardiomyocytes and heart tissue were extracted with ice‐cold lysis buffer and centrifuged at 12,000 × g for 10 min., and the resultant supernatant assayed using BCA protein assay kit standardized to BSA. Equal amounts of total protein (40 μg) were loaded, separated by SDS‐PAGE, and transferred to polyvinylidene fluoride (PVDF) membrane. The membranes were blocked in 5% TBST fat free milk (blocking buffer) for 2 hrs, briefly washed, and then incubated overnight at 4°C with specific primary antibodies against mTOR (1:2000), phospho‐mTOR (1:1000), Beclin‐1 (1:1000), Bcl‐2 (1:2000), Bax (1:2000), Caspase‐3 (1:2000), or LC3B (1:2000). Blots were then incubated in anti‐rabbit secondary antibody (1:10,000) for 2 hrs. Proteins were detected using a chemiluminescence system, according to the manufacturer's instructions. Band intensities were quantified using Imaging System Analysis software (VersaDoc Mp5000; Bio‐Rad). Relevant band intensities were quantified after normalization to the amount of GAPDH protein as a positive control.

### Statistical analysis

All values are expressed as the mean ± S.D. Comparisons between multiple‐group means were performed using one‐way anova. Multiple comparisons between the groups were performed using Least Significant Difference (LSD) method. *P*‐values <0.05 were considered to be statistically significant. All statistical analyses were performed using SPSS version 17.0 (International Business Machines Corp., Armonk, NY, USA).

## Results

### DSS protects against cardiomyocyte H/R injury

A dose of DSS under 10 μM was used in these experiments, as we previously reported 32. The viability of cardiomyocytes was significantly reduced after H/R (6 hrs of hypoxia followed by 18 hrs of reoxygenation) when compared to the control group. Danshensu pre‐treatment almost completely restored survival of cardiomyocytes exposed to H/R. However, addition of Rap prevented this protective action of DSS. These data suggest that DSS treatment may protect against H/R induced cardiomyocyte cell death through mTOR signalling.

### DSS inhibits H/R induced autophagy

As autophagic flux is a dynamic process, the extent of autophagy cannot be evaluated only by autophagosome formation. Staining of mRFP‐GFP‐LC3 is a useful tool for evaluating the extent of autophagic flux, as it can differentiate between autophagosomes and autolysosomes 33, 34. There is a pH difference between the acidic autolysosome and the neutral autophagosome. A green fluorescent protein GFP signal is quenched in an acidic environment. By contrast, a red fluorescent protein RFP signal is more stable in an acidic environment 33, 35. Therefore, autophagosomes are labelled yellow (green dots and red dots overlay in merged images), while autolysosomes are labelled red. Thus, autophagic flux can be monitored by detecting and analysing the two different fluorescent signals.

**Figure 2:**
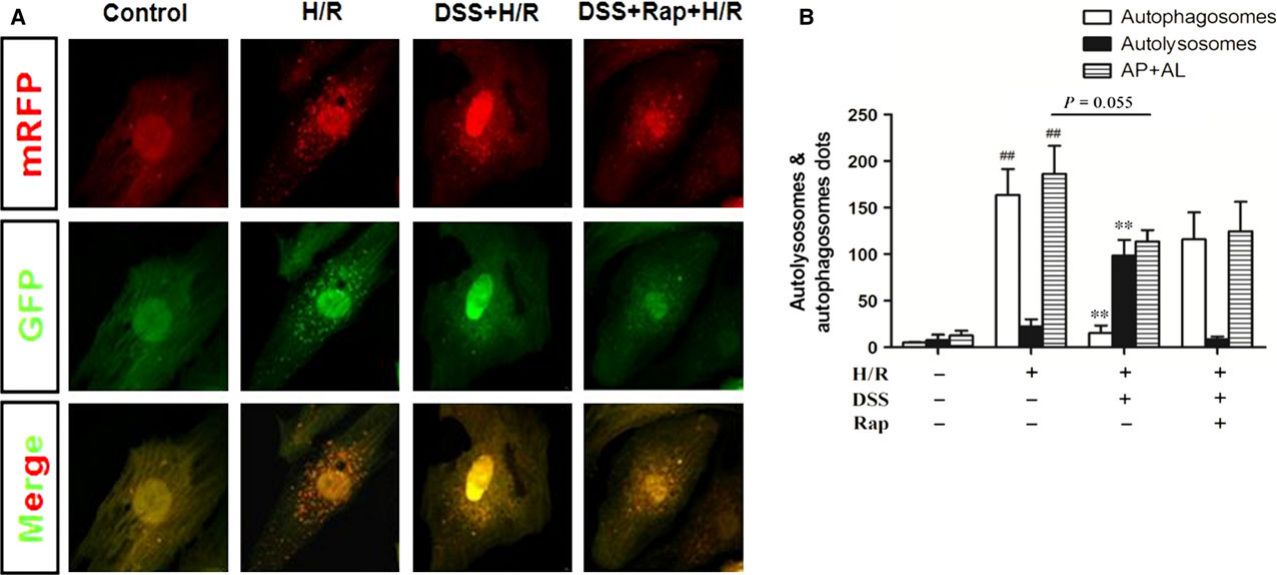
Effect of DSS on Autophagy flux in cardiomyocytes. (**A**) Representative immunofluorescence images of NRCMs expressing mRFP‐GFP‐LC3 adenovirus for 36 hrs. (**B**) Quantitative analysis of autophagosomes (AP, white bars), autolysosomes (AL, black bars), and both (AP+AL, grey bars) in NRCMs. Representative of *n* = 3 experiments. Data are expressed as the mean ± S.D. ^##^ *P* < 0.01 *versus* Control group. **P < 0.01 *versus* H/R group.

Infection with the mRFP‐GFP‐LC3 adenovirus resulted in successful expression of the fluorescent proteins (Fig. 2A). Neonatal rat cardiomyocytes (NRCMs) cultured in a normoxic environment displayed basal autophagic flux. However, H/R increased the numbers of autophagic structures, which were predominantly autophagosomes, without an increase in autolysosomes. Thus, H/R injury induced autophagy and disturbed autophagic flux in NRCMs. There was an increase in red puncta and a decrease in yellow puncta in the DSS+H/R group compared to the H/R group (Fig. 2B), suggesting that DSS attenuated autophagosome formation in NRCMs. The attenuation effect of DSS on autophagosome formation was abolished by Rap. These data suggest that DSS inhibits autophagosome formation under H/R injury conditions.

### Inhibition of autophagy and apoptosis by DSS involves activation of mTOR signalling

**Figure 3:**
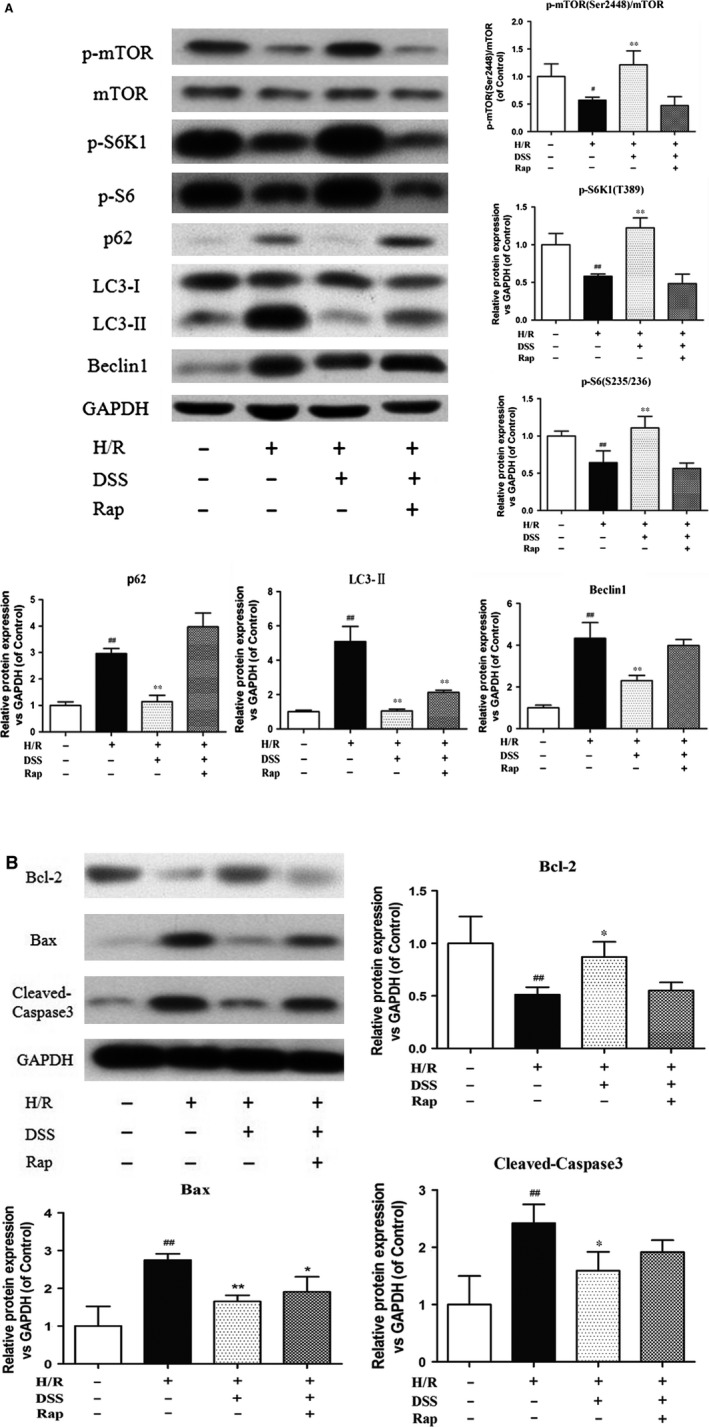
Effects of DSS on autophagy and apoptosis related gene expression in cardiomyocytes. (**A**) Western blotting results of inhibition of autophagy‐related gene expression by DSS. (**B**) Western blotting results of inhibition of apoptosis‐related gene expression by DSS. Data are expressed as mean ± S.D., *n* = 3. ^#^ *P* < 0.05, ^##^ *P* < 0.01 *versus* Control group. *P < 0.05, **P < 0.01 *versus* I/R group.

**Figure 4:**
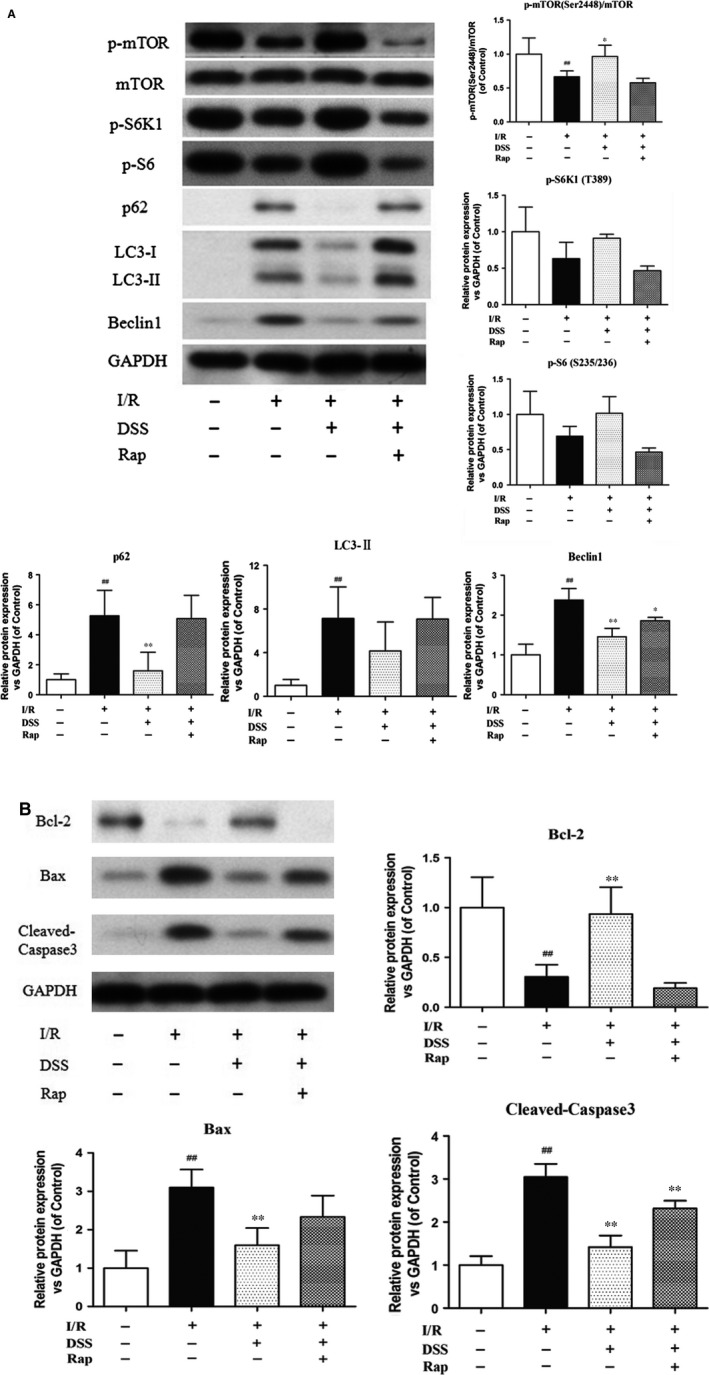
Effects of DSS on mTOR‐mediated autophagy and apoptosis in isolated rat hearts. (**A**) Western blot results of inhibition of autophagy by DSS. (**B**) Western blotting results of inhibition of apoptosis by DSS. Data are expressed as mean ± S.D., *n* = 3. ^##^ *P* < 0.01 *versus* Control group. *P < 0.05, **P < 0.01 *versus* I/R group.

**Figure 5:**
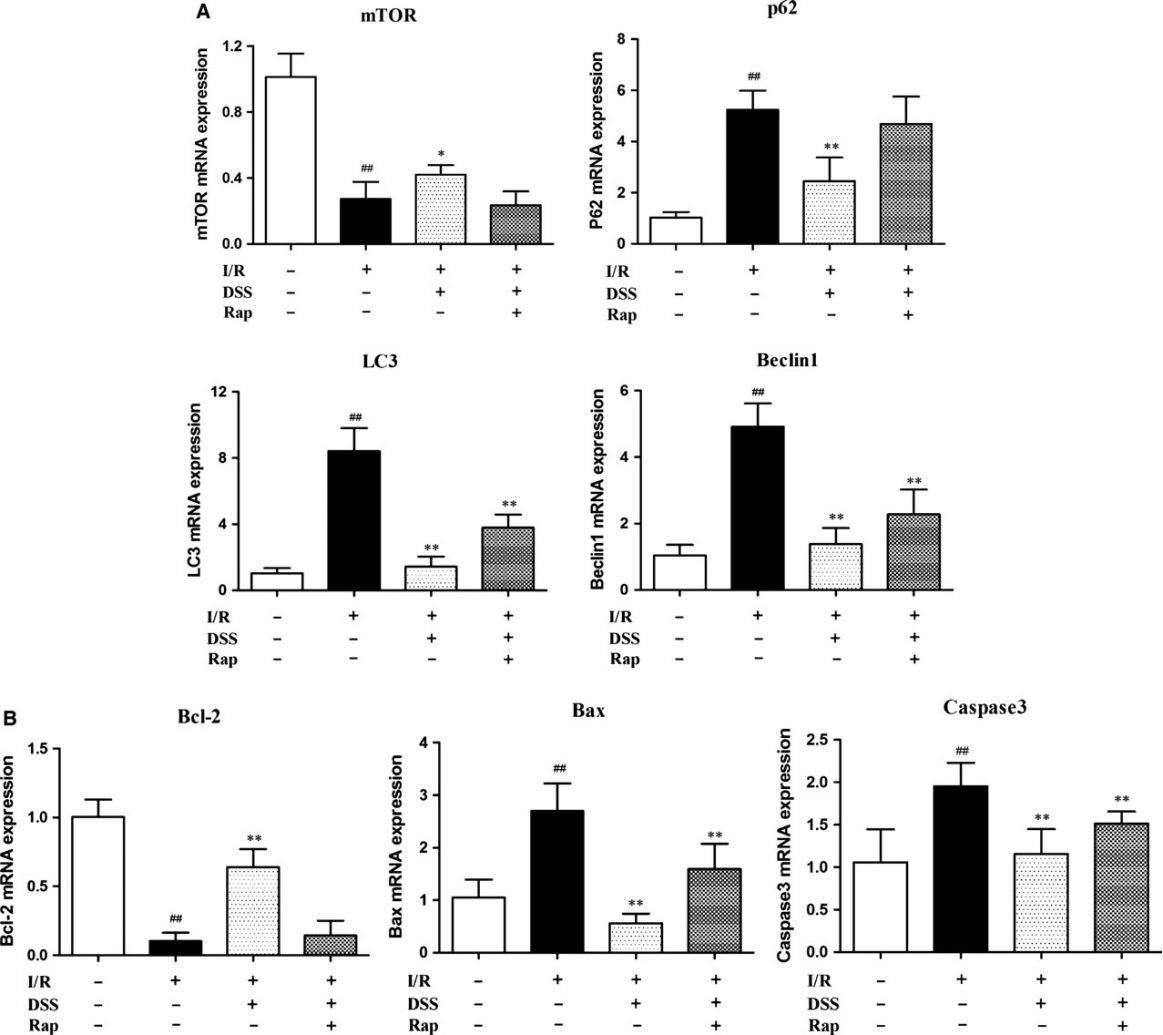
Effects of DSS on mTOR‐mediated autophagy and apoptosis in isolated rat hearts. (**A**) Evaluation of autophagy‐related gene expression after 60 min. reperfusion by RT‐PCR analysis. (**B**) Evaluation of apoptosis‐related gene expression after 60 min. reperfusion by RT‐PCR analysis. Data are expressed as mean ± S.D., *n* = 6. ^##^ *P* < 0.01 *versus* Control group. *P < 0.05, **P < 0.01 *versus* I/R group.

To explore the molecular mechanisms by which DSS protects against I/R injury, we assessed autophagy and apoptosis pathways by western blot analysis in cardiomyocytes and isolated hearts (Figs 3 and 4). Cardiomyocytes and myocardial samples taken from the left ventricle after pre‐treatment with DSS showed a significant increase in mTOR phosphorylation and the phosphorylation levels of S6K and S6, the downstream targets of mTOR, compared to the I/R group. Addition of Rap prevented this increase with DSS treatment. Hypoxia/reoxygenation or I/R injury increased protein expression of the autophagy markers p62, LC3‐II and Beclin‐1, and the apoptosis markers Bax and Caspase‐3. Hypoxia/reoxygenation or I/R injury also reduced expression of the anti‐apoptosis protein Bcl‐2, which was blocked by the DSS treatment. Similar patterns were observed in isolated rat hearts by RT‐PCT (Fig. 5). These data provide additional evidence that DSS treatment results in mTOR activation, which is accompanied with attenuated autophagy and apoptosis during I/R injury.

### DSS restores cardiac function and protects isolated rat hearts from I/R injury

**Figure 6:**
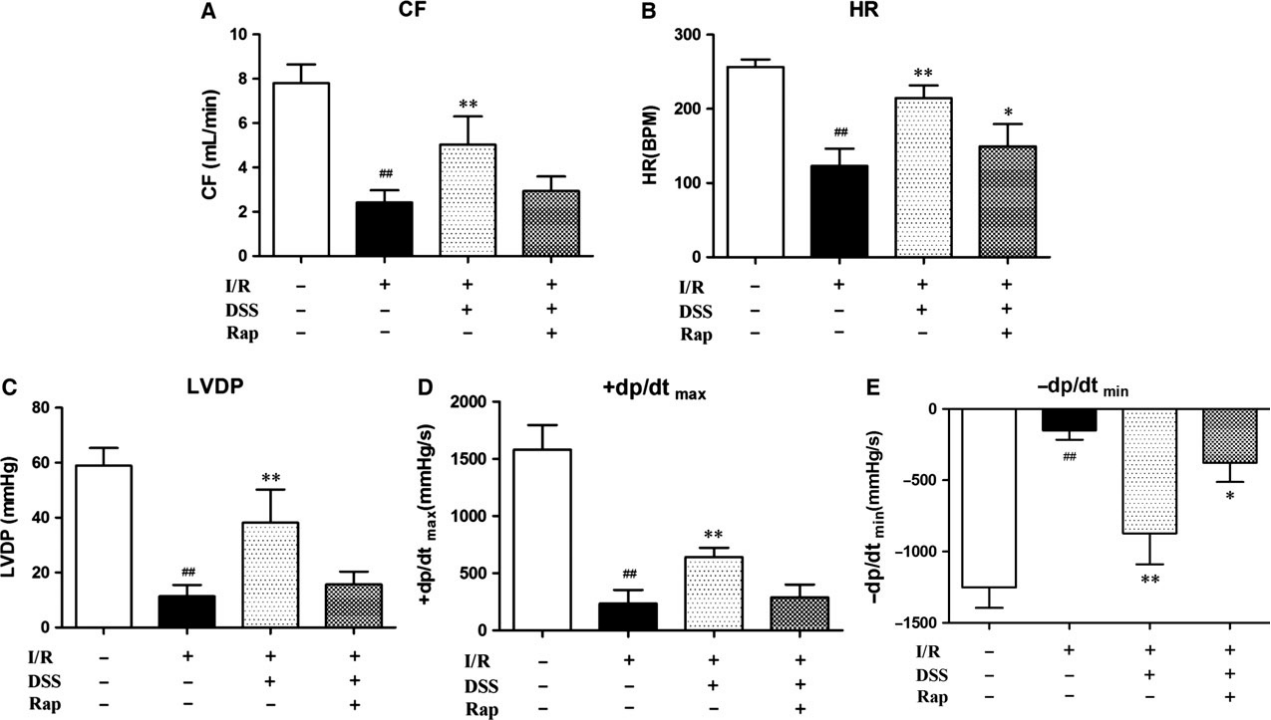
Effects of DSS on cardiac function in isolated rat hearts. The hearts underwent 20 min. of stabilization, 30 min. of ischaemia, and 60 min. of reperfusion. Drugs were administered before ischaemia for 10 min. (**A**) Coronary flow (CF). (**B**) Heart rate (HR). (**C**) Left ventricular developed pressure (LVDP). (**D** and **E**) Maximal and minimum rate of pressure development (±dp/dt max), respectively. All parameters were measured at the end of the reperfusion period. Data are expressed as mean ± S.D., *n* = 6. ^##^ *P* < 0.01 *versus* Control group. *P < 0.05, **P < 0.01 *versus* I/R group.

The analysis of hemodynamic parameters on cardiac function is shown in Figure 6. Compared to the Control group, the CF, HR, LVDP and ±dp/dt max at the end of the reperfusion period were significantly decreased in the I/R group. Pre‐treatment with DSS resulted in improved cardiac function after I/R, with a significant increase in CF, HR, LVDP and ±dp/dt max compared to the I/R group. These effects of DSS were blocked by addition of Rap. These data suggest that DSS can protect cardiac function against I/R injury by activation of mTOR signalling.

### Protective effect of DSS on CK and LDH release in coronary effluents

**Figure 7:**
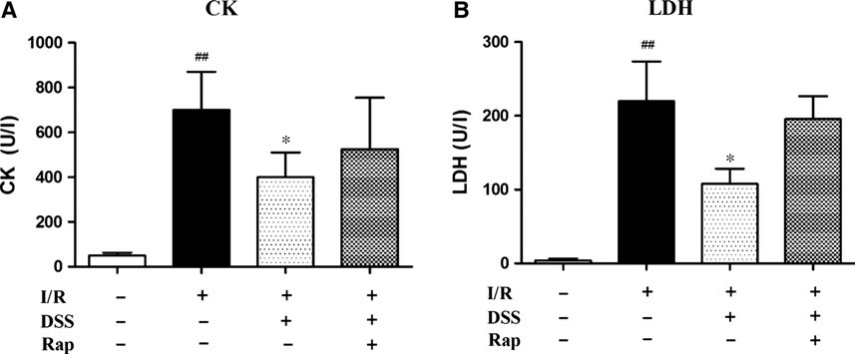
Effects of DSS on injury biomarkers in coronary effluents. (**A**) Creatine kinase (CK). (**B**) Lactate dehydrogenase (LDH). Data are expressed as mean ± S.D., *n* = 6. ^##^ *P* < 0.01 *versus* Control group. *P < 0.05 *versus* I/R group.

Expression of CK and LDH were significantly increased at the end of reperfusion in the I/R group. Pre‐treatment with DSS at 10 μM for 10 min. before ischaemia significantly decreased CK and LDH release, while this effect was suppressed by Rap treatment (Fig. 7). These data confirm that DSS protected the heart from I/R injury by activating mTOR signalling.

## Discussion

In the present study, we found that ischaemia‐induced cardiac dysfunction and cell death were attenuated by mTOR activation following DSS treatment. Furthermore, cell death and cardiac dysfunction occur concomitantly with upregulation of autophagic activity in ischaemic cardiomyocytes. Thus, even though cardiomyocyte autophagy is upregulated as an adaptive‐response mechanism, the observed increase in autophagosome formation during I/R suggests an impaired autophagosome clearance, which may paradoxically contribute to cell death and myocardial injury 7. We provide novel evidence that DSS may reduce autophagy during I/R by upregulating mTOR signalling and reducing autophagosome formation. Thus, blocking post‐ischaemic autophagy by DSS can prevent excessive ischaemic myocardial injury and improve cardiac function. We confirmed that the cardioprotective effect of DSS was due to activation of mTOR by treating NRCMs with Rap to irreversibly cause inhibition of mTOR. Overall, our findings suggest a previously unknown cardioprotective effect of DSS through mTOR activation and the subsequent inhibition of ischaemia‐induced autophagy and apoptosis.

As a typical serine/threonine protein kinase that plays a vital regulatory role in autophagy and apoptosis during ischaemic injury, mTOR is essential for cardiac structure and function 36, 37, 38. Dysregulation of mTOR activity has been implicated in a wide range of pathologic conditions 39. We found that mTOR activity was suppressed in response to I/R, which was a crucial step for autophagic cell death in cardiomyocytes. Although autophagy is thought to play a central role in regulating cell death and survival, our data suggest that excessive autophagy, such as increased formation of autophagosomes and autophagic flux can lead to cardiomyocyte cell death 40. Despite our finding that DSS activation of mTOR can reduce ischaemia‐induced autophagy and tissue injury, it remains unclear whether continuous mTOR activation acts as an endogenous survival signalling mechanism.

The features of cardiac autophagy and apoptosis occurred concurrently with decreased CF, HR, LVDP and ±dp/dt max during I/R injury. We suggest that the function of autophagy in cardiomyocytes may be stimulus dependent, and that autophagy may be a potential prognostic marker for the pathogenic state of the heart and thus useful for cardiovascular disease management.

Another major finding of our study was the upregulation of apoptotic proteins and autophagy‐related proteins including Beclin‐1, LC3 and p62 during I/R. The expression of these proteins was decreased following DSS treatment, suggesting that DSS negatively regulates autophagy. We also found that DSS markedly alleviated ischaemia‐induced cardiomyocyte apoptosis, with decreased expression of Bax and Caspase‐3, and increased expression of the anti‐apoptotic protein, Bcl‐2. Furthermore, these effects of DSS were blocked by Rap pre‐treatment, suggesting that mTOR activation underlies the mechanism of DSS inhibition of ischaemia‐induced cell death.

Our findings of down‐regulation of LC3, Beclin‐1, p62, Bax and Caspase‐3, and up‐regulation of Bcl‐2, following DSS treatment suggest a potential cross‐talk between autophagy and cell death in ischaemic conditions. Notably, the therapeutic effects of DSS were previously attributed to its inhibitory role on the apoptotic pathway. Our data suggest that DSS targets both autophagic and apoptotic pathways *via* mTOR activation. We found that mTOR activation correlated with Bcl‐2 stimulation and down‐regulation of pro‐apoptotic proteins. Of note, Bcl‐2 binds to and inhibits BH3 domain of Beclin‐1 that is crucial for autophagic process 22, 41. Thus, activation of mTOR is an important mechanism for DSS to regulate apoptosis and autophagy.

Danshensu, a water‐soluble constituent of *S. miltiorrhiza* (Danshen), is well‐recognized for its cardiovascular activity 42. Danshensu was previously reported to protect the heart against I/R injury by reducing ROS generation and inhibiting cell apoptosis 25, 26, 27. However, the effects of DSS on the autophagy and apoptosis pathways during I/R injury are unknown. In this study, we demonstrated that DSS protects against I/R injury by inhibiting excessive autophagy. Since autophagy can be regulated by mTOR, it was imperative to examine whether DSS inhibition of autophagy is due to mTOR activation. Regarding this, we treated NRCMs with Rap to irreversibly inhibit mTOR expression. Further readout of mTOR activity by determining the phosphorylation levels of downstream effectors of mTOR; S6K and S6 provided evidence to conclude that the mechanism through which DSS inhibits excessive autophagy and apoptosis is through mTOR activation.

In summary, the present study revealed a new mechanism underlying the cardioprotective effect of DSS, in which mTOR activation decreased the activity of the autophagy pathway and concomitantly decreased apoptosis after ischaemia. Hence, DSS improves cardiac function through inhibition of apoptotic and autophagic mechanisms. Further studies examining the association between regulatory pathway of autophagy and cardioprotective effects of mTOR activators may provide novel therapeutic strategies for cardiovascular disease management.

## Conflicts of interest

The authors confirm that there are no conflicts of interest.
